# Effects of stretching and warm-up routines on stability and balance during weight-lifting: a pilot investigation

**DOI:** 10.1186/1756-0500-7-938

**Published:** 2014-12-20

**Authors:** Rolf Adelsberger, Gerhard Tröster

**Affiliations:** Federal Institute of Technology (ETH), Zurich, Gloriastrasse 35, Zurich, 8092 Switzerland; Institute for Electronics, Gloriastrasse 35, Zurich, 8092 Switzerland

**Keywords:** Force Sensor, Wearable, Weightlifting, Balance, Stretching, Analysis

## Abstract

**Background:**

The efficacy of warm-up and stretching in weight-lifting remains unknown, especially for the weight-lifter’s stability and balance during lifting.

**Methods:**

13 subjects were randomly assigned a 10-minute stretching routine (SR) or a 10-minute warm-up routine (WR) and compared against 5 controls (no stretching or warm-up). Before and after the individually assigned routine, the participants’ centre of pressure (CoP) was assessed using plantar-pressure sensors. The subjects were measured during 10 repetitions of air squat (no load, “AS”), front squat (FS; 20 kg/15 kg bar), overhead squat (OHS; m: 20 kg / f: 15 kg bar), and a deadlift lifting exercise (“DL”; 20 kg/15 kg bar). The impact on CoP dynamics of the warm-up and stretching routines were examined with repeated two-factor analysis of variances (ANOVA) of the mean and the coefficient of variance (CV, shown in %), as proxies for stability and balance.

**Results:**

After stretching, the SR athletes shifted the mean CoP towards the toes (≈1 cm; p < 0.01) while the WR athletes shifted the CoP towards the heels (≈1 cm; p < 0.01) during AS. For the remaining exercises, the SR athletes shifted the CoP towards the heels (between 0.8 cm and 5.7 cm) compared to WR (≈1.9 cm towards the heels in FS, no significant change in OHS (≈1 mm) and DL (≈3 mm)). The controls did not show any change between pre- and post-datasets. After stretching, the CV decreased for the AS and OHS exercises (AS: 10.2% to 7.0%, OHS 9.8% to 7.8%), but increased after WR (AS: 7.1% to 10.1%) or did not change significantly (OHS). Both WR and SR resulted in increased CV values for FS and DL. No change of CV was observed in the controls.

**Conclusions:**

SR had a stronger impact on CoP during the assessed exercises than either WR or controls. A reduction in CV after SR exercises (AS, OHS) suggests a clear improvement in stability and balance during weight-lifting. The lack of a significant effect for complex movements (OHS) suggests only a limited effect of a 10-minute warm-up routine on CoP features. 10 minutes stretching might therefore be more efficient for improving stability than a general 10 minute warm-up.

## Background

**Figure 1:**
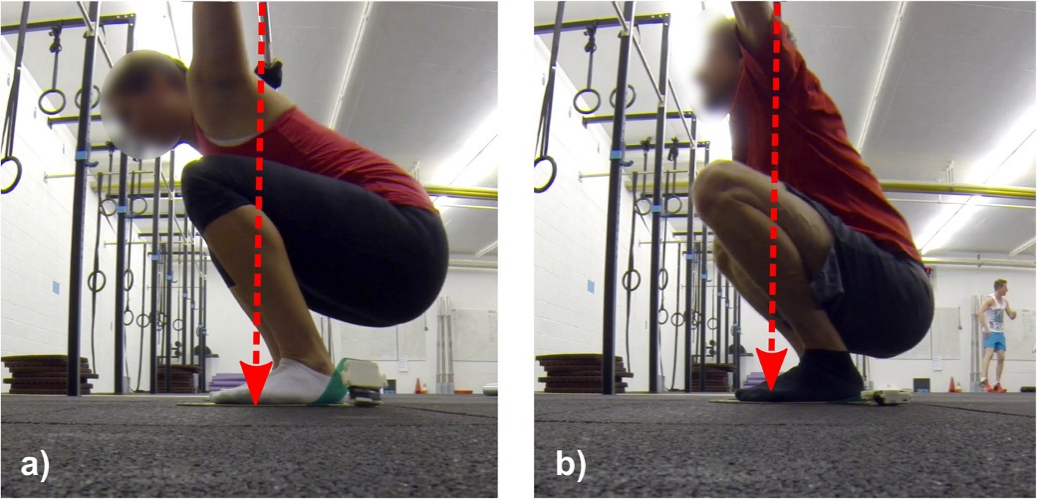
**Inflexible and flexible athlete: Comparing the squats from two athletes with different RoM.** The red arrow represents the gravitational force from the barbell. **a)** Example of a limited-RoM squat; **b)** a squat with good RoM.

The purpose of warm-up (WR) and stretching (SR) routines is to increase the range of motion (RoM) of skeletal muscles and the associated connecting tissue surrounding the joints, and thus to improve RoM of the exercise-specific kinematic chain [1–3]. An improvement in range of motion enables athletes to adopt more optimal positions during weight-lifting and thus to exploit the muscular/strength capabilities [4–6]. The concept is clearly demonstrated in Figure 1, where the athlete needs to extend her arms further backwards in order to achieve a balanced posture (Figure 1a). The result is increased torque in various parts of her body, especially the shoulder joint [7]. In contrast, the athlete in Figure 1b showed a superior RoM and was thus able to maintain a bio-mechanically more optimal position. On the other hand, negative side effects such as a reduction of peak force up to 8% have been demonstrated to be caused by static stretching [8]. As weight-lifting athletes are cautious not to trigger detrimental effects to their maximal strength, static stretching is often avoided [9, 10]. Alternative techniques such as dynamic stretching, proprioceptive neuromuscular facilitation, and self-myofascial release have been shown to not affect peak strength negatively whilst bearing the positive effects of static stretching [11–13]. It has also been shown in related work that stretching can improve the balance performance of athletes [14]. Athletes with good stability seem to be able to control the barbell better in extreme poses, e.g. in the bottom of an (overhead) squat. However, elite athletes often fail lifts in extreme positions (e.g. at the bottom of a squat) due to a lack of balance [15]. Thus, stability and balance seem to be key parameters for control during weight-lifting and for successfully performing advanced lifting exercises. However, the role of stretching or warming-up prior to lifting exercises, especially on the stability of the centre of pressure and the subject’s balance during lifting, remains unknown. It is plausible that despite playing a positive role in enhancing the range of motion, negative side effects regarding stability and balance are caused by warm-up or stretching.

Analysis of centre of pressure (CoP) data acquired from plantar pressure sensors allows dynamic postural parameters e.g. balance to be estimated. Data from such approaches also suggest that a subject’s CoP is correlated to changes in RoM [16, 17]. The goal of this study was to compare general warm-up with stretching that is focused on joints and muscles that are involved in basic weightlifting exercises in order to assess the role warm-up or stretching on static and dynamic measures of stability and balance during weight-lifting. An additional aim of this study was to evaluate the applicability of the sensor system in weightlifting settings.

Three questions were asked in this study:

- Does stretching alter features of CoP?
- Does moderate warm-up alter features of CoP?
- Are there differences between the effects of warm up and stretching for stability during weight-lifting?

## Methods

**Figure 2:**
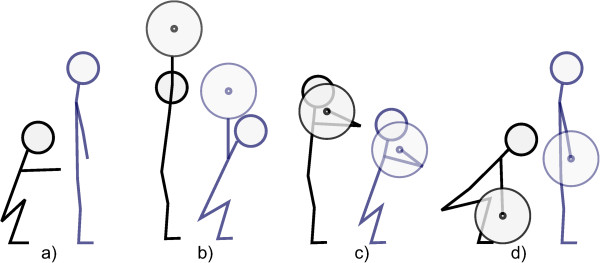
**The four exercises: different colours represent different key-poses that have to be reached.** To successfully perform an exercise an athlete is required to start in the black pose, reach the blue pose and finish in the black pose again. **a)** Air Squat (AS); **b)** Overhead squat (OHS); **c)** Front squat (FS); **d)** Deadlift (DL).

In this study, several exercises were analysed. The air squat (AS, Figure 2a) is a squat without any external weight. In the overhead squat (OHS, Figure 2b), an athlete balances a barbell above his head with straight arms while performing a squat. During the front squat (FS, Figure 2c), the athlete has the barbell on his shoulders. The deadlift (DL, Figure 2d) is not a squat exercise since the barbell starts from the ground and finishes at hip height. However, in this study, an aim was also to understand the differences between warm-up and stretching routines on CoP features during different lifting exercises.

### Subjects

Thirteen athletes (mean age 28.2 ± 5.9 yrs) from a local functional training facility volunteered to be analysed in this study and underwent either stretching or warm-up exercises. In addition, 5 subjects were tested without stretching or warm-up exercises in order to provide a control. Inclusion criteria were an age between 18 and 75, no medical issues affecting their physical performance, and no prior training on the day of testing. The study was approved by the Ethics Committee of the ETH Zurich, Switzerland, for which all participants signed a form of consent in order to participate. All athletes were also required to have non-pathological RoM for the tested exercises; the facility performs functional movement screen (FMS) for each athlete at the date of the sign-up to identify deficiencies in RoM [18]. The subjects’ age, gender, and experience level (“novice”, “proficient”, “expert”) were all recorded. Male athletes used a 20 kg barbell, while female athletes were provided with a 15 kg barbell.

### Pressure sensor measurement system

**Figure 3:**
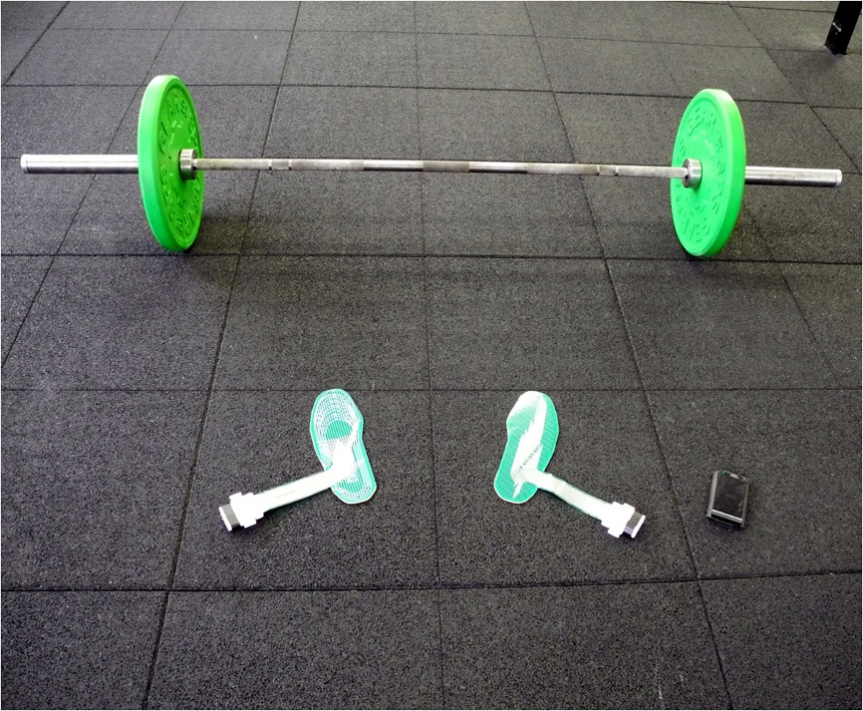
**Study Equipment.** Barbell with weights (40 kg); two sensor systems on plywood for left and right foot; smart phone controlling sensor systems and displaying data.

A wearable, non-obtrusive sensor system was used that is able to capture dynamic movement and plantar pressure data (Figure 3) [19]. The system was validated in prior work where it was shown to provide valid estimations on subjects’ balance performance [20, 21].

**Figure 4:**
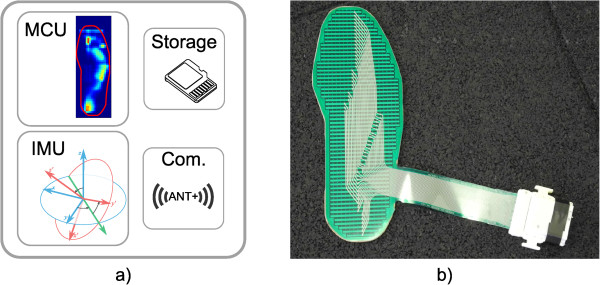
**Sensor system components. a)** MCU processes force readings; IMU samples inertial data; storage on SD card; wireless communication with ANT + protocol. **b)** Close-up of the sensor system on plywood.

**Figure 5:**
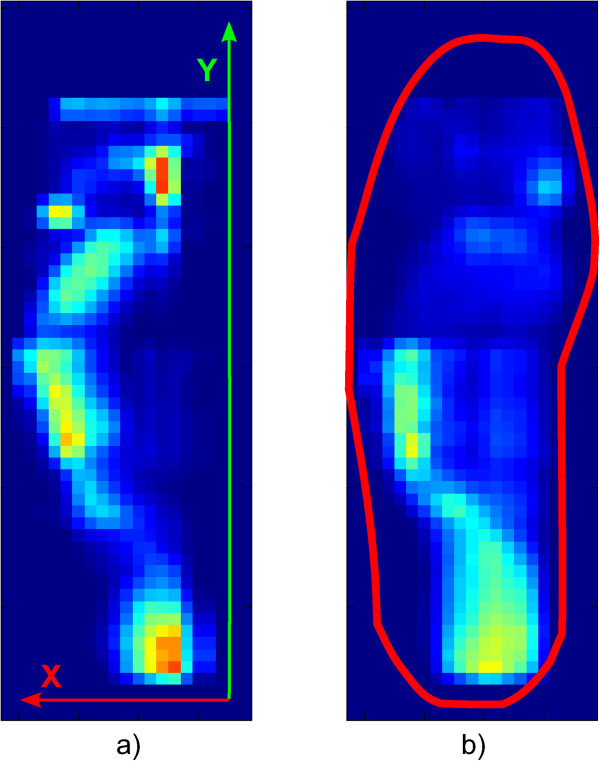
**Force-data illustrations: clearly visible the different levels of flexibility of the two individuals.** The subject on the right has a reduced area of the feet to be used for the exercise. Both images show left feet. **a)** Mean pressure map of a flexible subject for the initial 10 AS. Data axes are also included in this picture. **b)** Mean pressure map of a less-flexible subject for the initial 10 AS. Contour of sensor sole is shown in red.

The system was comprised of a thin and flexible foot-shaped plastic foil containing 1260 force-sensitive resistors (FSR) (Figure 4). A raw sensor sample featured 21 sensing points in the x-direction and 60 sensing points in y-direction (Figure 5). It has been validated against commercial plantar-pressure sensing systems [20]. Although the system is able to detect small differences between different shoe models [19], in this study, the sensor foil was glued onto a flat plywood surface for measurements (Figure 4b). In so doing, the impact of differences in shoes or feet sizes of the various subjects could be removed and the data could be acquired without individual bias. For this study, “zero-drop” shoes i.e. shoes with no drop from heels to toe, or only wearing socks was required from all subjects. During the tests, the athletes stood on the foil in socks or in their own (“zero-rise”) shoes. The sensor system sampled FSR values and concurrently recorded motion data from an inertial measurement unit (IMU). IMU data consisted of three-dimensional acceleration, rotation rate, and compass values. Accelerometer readings from the IMU were used to segment the data during analysis. At 100Hz, the system calculated the CoP of each foot and stored it locally.

In advance of the testing, the pressure measurement system was presented to each subject and all procedures were explained. Firstly, subjects were asked to perform 10 squats without additional weight (air squat, AS), followed by 10 overhead squats (OHS) with the assigned weight, and 10 front squats (FS) with the same weight. Finally, every subject performed 10 deadlifts (DL) (Figure 2). A coach of the facility supervised the correct execution of the exercises.

**Figure 6:**
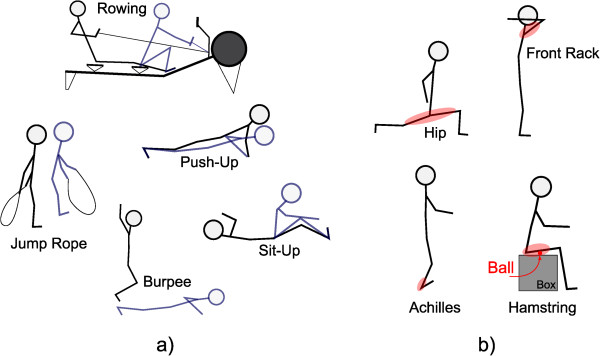
**Warm-Up and Mobility routines. a)** warm-up routines; **b)** mobility routines – treated musculotendinous units are highlighted in red.

After a 10 minute rest, each subject was then randomly assigned to perform the warm-up routines (WR), the stretching routines (SR) (Figure 6) or simply to further wait (control, CTR). Stretching exercises combined dynamic stretching routines [11], self-myofascial release (SMR) techniques [12], and proprioceptive neuromuscular facilitation [13] (Figure 6b). The warm-up routines consisted of a combination of exercises commonly used in the functional training facility (Figure 2). The subjects were asked not to go into exhaustion during warm-up. The CTR group was asked to wait 10 minutes sitting or standing. After the 10 minutes, each exercise was recorded once again. Between exercises, the subjects performed several steps to pick-up the barbell or to readjust stance etc. The movements were visible in the force data and also in accelerometer data.

All data were analysed using the Matlab software (R213b, MathWorks, Natick, MA). IMU and pressure data were segmented into episodes of AS, OHS, FS, and DL. Data intervals were labelled with the appropriate exercises. If an athlete performed unexpected movements during data acquisition, the sample was annotated to avoid false labels. For each interval of AS, OHS, FS, and DL, the algorithms extracted the features from pressure data. Since every subject had a different baseline level of mobility, and because feet sizes were different, all data were normalised to feet sizes. Here, in a pre-processing step, the region of interest for each data sample was extracted, i.e. the parts of the insole where the feet were standing and CoP coordinates were mapped to a common range ([0,1]).

For both feet, the centre of pressure was extracted: CoP_L_ and CoP_R_. The left and the right CoP were then combined into a single CoP for the whole body. We calculated the following features from CoP: mean, and the coefficient of variance (CV). All features were calculated on both dimensions, i.e. x and y (Figure 6), in a 300 ms sliding window with 50% overlap. CV was calculated as the fraction of the standard deviation from the mean value of the sample, i.e. CV = σ/μ. This feature reflected how dispersed or scattered the CoP was for a given exercise, as a proxy for stability in balancing exercises. For exercises that required limited balancing (e.g. DL), the CV feature was used as a surrogate for the regions of each subject’s feet that were used to generate a reaction force. These features have been demonstrated to be valid indicators for balance, stability and body-weight distribution [21].

Prior to addressing the hypotheses, the samples from all groups (pre-stretching/warm-up/control) were tested by ANOVA to examine whether statistically significant differences in the data sets were present. Based on all features, a three-factor analysis of variance (ANOVA, (alpha = 0.05, factors = [age, gender, exp.])) was performed to work out if age, experience or gender had a measurable effect on the data.

To answer the three questions listed in the first section, repeated two-factor ANOVA (α = 0.05, factors = [group{WR,SR,CTR} condition{pre,post}]) was used to detect statistically significant differences between pre- and post-routine data and to answer the three questions.

## Results and discussion

### Results

ANOVA did not reveal any significant differences in the CoP dynamics between any groups prior to stretching or warming-up. Video analysis showed that some individuals had superior flexibility than most of the others (Figure 1).

The control group (CTR) showed no significant changes between the first and the second tests (Tables 1 &2).

**Table 1:** **Development of the mean feature in the three groups**

Exercise	CTR	SR	WR
**AS**	30.5/30.4	36.8/39.0*	29.8/27.8*
**OHS**	28.0/28.2	36.7/32.6*	29.1/29.3
**FS**	30.1/30.1	35.7/24.2*	32.8/29.0*
**DL**	31.0/31.7	32.0/30.4*	28.6/27.6

The values represent insole coordinates (1 = 5 mm) and are presented as (pre/post) routine. Significant differences (p < 0.01) are denoted with an asterisk (*).

**Table 2:** **Development of the CV feature in the three groups**

Exercise	CTR	SR	WR
**AS**	9.6/10.6	10.2/7.0*	7.07/10.1*
**OHS**	16.2/14.6	9.8/7.8*	8.7/7.8
**FS**	11.9/9.6	9.2/14.8*	5.0/11.5*
**DL**	8.2/7.7	4.8/7.9*	4.1/7.8*

The values represent insole coordinates (1 = 5 mm) and are presented as (pre/post) routine. Significant differences (p < 0.01) are denoted with an asterisk (*).

The stretching routines affected the CoP for all exercises (Tables 1 &2). For AS, the mean CoP coordinates shifted approximately 11 mm towards the toes. For the OHS (20.5 mm; p < 0.01), FS (55.5 mm; p < 0.01) and DL (8 mm; p < 0.01) exercises, the mean CoP shifted towards the heels (Table 1). The coefficient of variation (CV) was also affected for all exercises by the stretching protocol. For AS and OHS the CV value decreased significantly (10.2% to 7.0%, and 9.8% to 7.8%, resp.), for FS and DL it increased (9.2% to 14.8% and 4.8% to 7.9%) (Table 2).

The warm-up routines affected AS and FS significantly on all features (Tables 1 &2). WR did not affect the OHS, and only weakly (p < 0.05) affected the CoP dynamics for DL. After warm-up, the mean CoP during AS shifted 10 mm towards the heels. For the FS exercise, the mean CoP was shifted approx. 19 mm towards the heels. The mean CoP for OHS remained unaltered (change of approx. 1 mm) and the effect on DL (approx. 5 mm) was only weakly significant (p < 0.05). For the AS exercise, the CV increased from 7.1% to 10.1%, for the FS exercise it increased from 5.0% to 11.5% and for DL, CV increased from 4.1% to 8.0%. CV was not affected significantly by WR for the OHS exercise. The differences in post-routine performances were significant for all exercises for the mean feature. The differences in the CV feature were only significant for AS and FS exercises, but not for OHS and DL.

### Discussion

Warm-up and stretching routines affect dynamic and static properties of the CoP during weight-lifting activities. However, stretching seems to increase stability in complex exercises (OHS) while warm-up does not affect these exercises. In other exercises, the effects of warm-up and stretching are comparable in terms of affecting CV and mean. As there was no effect present from the exercises alone, the detected changes in the WR and SR groups were likely to be caused by warm-up or stretching respectively.

The data from our study suggest that stretching plays a significant role on features of CoP. Common guidelines received by athletes from coaches are to try to keep the major portion of their body weight on the heels during the presented movements [22]. After stretching, the athletes were able to shift their mean CoP closer to the heels for OHS, FS and DL, but not for the AS exercise. While the reason for the shift in CoP towards the toes for the AS is unclear, it is possible that the change resulted from the SR athletes performing the second set of AS faster than the first (as confirmed by video footage). We did not control the speed at the time of the data acquisition. One possible reason is that AS performed rapidly might resemble a jump regarding muscle activation, i.e. AS became more quadriceps-driven. However, it is clear that this effect should be investigated in a different study.

The OHS exercise became more stable after the stretching exercises compared to pre-stretching (decreased CV). In combination with the mean CoP shifting to the heels, we believe that this was caused by a more upright posture that enabled the athletes to maintain the centre-of-mass (of the barbell) farther back and thus did not have to “fight” against the weight [23]. The OHS was the most challenging exercise in terms of flexibility, stability and balance. Thus an improvement in stability and balance is a strong positive result for stretching. Post-stretch, the athletes became less stable during the FS exercises, as indicated by an increased CV value. We believe that this is caused by an increased flexibility in muscles e.g. triceps, possibly limiting a proper technique in the front squat, while still presenting restrictions in other MTUs, e.g. the hip flexors. This would allow the athletes to maintain the mean CoP closer to the heels during the major part of the move, but it would pull them forward, at, e.g., the bottom of the squat. This hypothesis should be investigated in future studies. An analogous reasoning could explain the increased CV value during the DL exercises.

Regarding question 2: WR significantly affected CoP features during AS, FS and DL, but not during OHS (see Table 1 &2). For AS and FS exercise, there was a significant shift of the mean CoP to the heels. This shift might have been caused by multiple factors, for example adapted muscle activation or flexibility changes of, e.g., Achilles and hip flexors (AS) and additionally triceps (important for FS). We don’t think that a practice effect or muscle fatigue are a valid explanation for the observed shift, as the barbell weights used were light for all athletes and the exercises were simple and not new to the athletes. There was no significant shift of the mean CoP during OHS or DL. The overhead squat exercise did not benefit from WR, regarding CV. The CV increased between pre- and post WR during AS, FS and DL. This could be caused by decreased stability or by increased recruitment of plantar area. Because there was no significant increase in CV between pre- and post-testing of the OHS, an increase in plantar area recruitment is more likely. The OHS exercise is the most challenging regarding stability and balance, thus, if WR reduced stability we would have expected to find this effect also (especially) in the analysis of OHS. However, the CV feature for the OHS exercise was not affected significantly, and a trend towards increased stability was observed.

Question 3 was more difficult to answer. There were statistically significant differences in most features after post-routine analyses between the stretching group and the warm-up group. The difference of the impact of both routines was significant for the feature mean during all exercises. The CV feature showed a statistically significant differences between the routines only for exercises AS and FS. Due to considerations regarding air squat presented above (i.e. possible change in speed), we refrain from interpreting the impact of both routines on AS. For the other three exercises, we derived that the impact of the stretching routines were significantly different from the warm-up routine regarding mean CoP. Relative changes of the mean feature from pre-intervention to post-intervention data are larger in the stretching group.

### Limitations

A large variability in baseline flexibility could introduce an unknown bias to the analysis. There were two athletes with an exceptionally good but an much lower overall flexibility. However, the reduced flexibility was still not considered pathological as assessed by the FMS tests. In the squat position, the flexible athlete was able to maintain an angle between the floor and his back of approx. 65 deg. The non-flexible athlete, however, achieved a maximal angle of 35 degrees. In future studies, the subjects should be assessed regarding their baseline flexibility and mobility prior to group assignments. Established systems exist to categorize the flexibility of athletes [18, 24], and these could be easily employed to enhance the quality of the data. Also the number of participants should be increased, especially if pre-screening is applied. In addition, all subjects should be monitored during the intervention: we suspected some participants of the warm-up group worked at a too high an intensity and therefore their post-intervention results (e.g. AS speed) were possibly biased. Here, video-analysis or motion capture could deliver data on the RoM of specific joints.

## Conclusions

We compared stretching routines with warm-up routines regarding their effects on CoP of four weight-lifting exercises. A plantar-pressure sensor system recorded data that was later analysed on a computer. Dynamics of the centre-of-pressure were used as a proxy for centre-of-mass dynamics. By analysing changes in CoP/COM dynamics, changes in stability, balance and COM distribution in the subjects were detected.

Both strategies affected features of CoP: mean centre of pressure values shifted to the heels during OHS and FS, which could indicate a more upright body posture. The changes in mean CoP during AS were contradicting, but we assume that the increased speed at which the stretching group performed the AS in the second set was causing those subjects to shift their weight to their toes rather than to the heels. The warm-up routines did not cause a statistically significant effect for the OHS exercise, whereas the stretching routines did. Stretching seemed to be beneficial regarding stability, as the CV decreased significantly between pre- and post-stretching testing during the most difficult exercise, the overhead squat.

A comparison of the effects of both routines was difficult and due to the aforementioned bias by speed etc. during AS. We don’t think that a reliable statement could be made about which routine is more advantageous for athlete’s balance/stability performance in AS. However, we think that there is evidence of an advantageous effect on stability of stretching, as the improvements in CV during OHS were significant.

### Outlook

A follow-up study should focus on one distinctive exercise. We propose to address OHS due to the requirements on balance/stability or FS due to its relatively low complexity, but significant requirements on lower-body flexibility. Furthermore, a focused view of one item in the kinematic chain, e.g. the ankle joint, seems to be appropriate due to the fact that ankle flexibility seems to be the most limiting factor for most athletes. Another interesting question is the role of the bar weight. It would be interesting how the balance/stability performance changes with increasing weight.

The used sensor system could also be applied as a training tool. CoP data can be visualized on a tablet or smart phone and displayed to an athlete in real time. A subject then could directly alter her body posture for a more optimal position.
